# Transcriptomic Analysis Reveals Panicle Heterosis in an Elite Hybrid Rice ZZY10 and Its Parental Lines

**DOI:** 10.3390/plants12061309

**Published:** 2023-03-14

**Authors:** Zhengzheng Zhong, Yawen Wu, Peng Zhang, Guocheng Hu, Dong Fu, Guoping Yu, Hanhua Tong

**Affiliations:** 1 State Key Laboratory of Rice Biology and Breeding, China National Rice Research Institute, Hangzhou 311400, China; 2 State Key Laboratory of Biocatalysis and Enzyme Engineering, School of Life Sciences, Hubei University, Wuhan 430062, China; 3 National Nanfan Research Institute, Chinese Academy of Agricultural Sciences, Sanya 572025, China

**Keywords:** rice (*Oryza sativa* L.), heterosis, transcriptome, photosynthesis, panicle length

## Abstract

Heterosis is the phenomenon in which some hybrid traits are superior to those of their parents. Most studies have analyzed the heterosis of agronomic traits of crops; however, heterosis of the panicles can improve yield and is important for crop breeding. Therefore, a systematic study of panicle heterosis is needed, especially during the reproductive stage. RNA sequencing (RNA Seq) and transcriptome analysis are suitable for further study of heterosis. Using the Illumina Nova Seq platform, the transcriptome of ZhongZheYou 10 (ZZY10), an elite rice hybrid, the maintainer line ZhongZhe B (ZZB), and the restorer line Z7-10 were analyzed at the heading date in Hangzhou, 2022. 581 million high-quality short reads were obtained by sequencing and were aligned against the Nipponbare reference genome. A total of 9000 differential expression genes were found between the hybrids and their parents (DG_HP_). Of the DG_HP_, 60.71% were up-regulated and 39.29% were down-regulated in the hybrid. Comparative transcriptome analysis revealed that 5235 and 3765 DG_HP_ were between ZZY10 and ZhongZhe B and between ZZY10 and Z7-10, respectively. This result is consistent with the transcriptome profile of ZZY10 and was similar to Z7-10. The expression patterns of DG_HP_ mainly exhibited over-dominance, under-dominance, and additivity. Among the DG_HP_-involved GO terms, pathways such as photosynthesis, DNA integration, cell wall modification, thylakoid, and photosystem were significant. 21 DG_HP_, which were involved in photosynthesis, and 17 random DG_HP_ were selected for qRT-PCR validation. The up-regulated PsbQ and down-regulated subunits of PSI and PSII and photosynthetic electron transport in the photosynthesis pathway were observed in our study. Extensive transcriptome data were obtained by RNA-Seq, providing a comprehensive overview of panicle transcriptomes at the heading stage in a heterotic hybrid.

## 1. Introduction

Rice (*Oryza sativa* L.) is a model cereal crop and one of the main global food crops. Heterosis is a phenomenon in which hybrids show superior performance to one or both parental phenotypes. In the past fifty years, heterosis has significantly increased the yield of global rice crops [1,2]. Hybrid rice varieties, such as Shanyou63 and Liangyoupei9, are widely cultivated in China. One of the best approaches for achieving super-high yields of rice is through inter-subspecific hybrids [1]. The yield of hybrid varieties of indica and japonica increased by approximately 25% [3]. Zhongzheyou 10 (ZZY10), which shows very strong yield heterosis in some agronomic traits and is widely adapted, is an elite rice hybrid released in 2016. Hybrid ZZY10 was developed using Zhongzhe A (ZZA) and Z7-10 as its two parental lines. The Cytoplasmic Male Sterile (CMS) line ZZA, which is widely used as the parent, has created several excellent hybrids cultivated in China. The most attractive feature of ZZY10 is strong yield heterosis, including panicle length (PL), 1000 grain weight (1000-GW), number of secondary branches (SBN), tiller number (TN), plant height (PH), and grain width (GW), which is termed optimal heterosis and is extremely required in breeding. This hybrid ZZY10 can be systematically used for studying the molecular mechanism of heterosis.

Improving photosynthesis is one of the most effective ways of increasing crop yield [4,5]. However, most previous studies analyzed improvements in leaf photosynthesis, but few have assessed non-foliar green tissue [6,7]. Panicles of rice and ears of wheat have strong photosynthetic capacities. Photosystem I (PSI) and photosystem II (PSII) protein complexes play a crucial role during photosynthesis [8,9]. All protein factors involved in the kinetic reactions of PSI and PSII complexes must be characterized in crops to improve photosynthetic efficiency and productivity [10,11]. It is speculated that improving ears photosynthesis in wheat will help increase the yield by 12–60% [12,13]. Therefore, improving photosynthetic efficiency in the ear is crucial for yield [14].

RNA sequencing-based transcriptomic analysis is a valuable strategy for exploring different pathways and regulatory networks and can help better understand biological processes [15,16,17]. Using transcriptome data as an informative platform, we can investigate the molecular mechanism of heterosis and the complex gene expression patterns associated with heterosis in crops (like rice, maize, and wheat) [18,19,20,21,22,23]. The classical genetic pattern of heterosis results in dominance, over-dominance, additivity, and epistasis [23,24,25,26].

In the present study, we concentrated on transcriptomic data from the Maintainer Line Zhongzhe B (ZZB), Z7-10, and ZZY10 to analyze the molecular aspects of heterosis. The analysis provides some explanations for the optimal heterosis of increased panicle length (PL) in hybrid ZZY10. The comparative transcriptomes of ZZB, Z7-10, and the hybrid ZZY10 were comprehensively analyzed to provide insights into genetic hypotheses, expression divergence, and the regulation pathway of genes between the parents and the hybrid. 

## 2. Results

### 2.1. Characterization of ZZY10 and Its Two Parental Lines 

ZZY10 is a rice hybrid produced by crossing the maternal line ZZB and the paternal line Z7-10. As suggested by Pickett [27], many yield-related traits of ZZY10 could show heterosis (Table 1). Interestingly, the panicle length of ZZY10 exceeded the panicle length of both parents (Table 2). We investigated the gene expression and phenotypes of ZZY10, ZZB, and Z7-10 using RNA-seq. The yield-related traits of ZZY10 showed heterosis (Table 1), as suggested by Pickett [27]. ZZY10 also has a longer panicle than its parents (Table 2). According to the phenotype of F_1_ hybrid ZZY10, mid-parent heterosis (MPH) and high-parent heterosis (HPH) were calculated to measure heterosis. The values of MPH and HPH are shown in Table 2. At the heading date, the MPH and HPH of the panicle length were 10.79% and −0.77%, respectively. In comparison, the MPH and HPH of the panicle length were 1.44% and −3.05% at the ripening stage, respectively (Table 2). The difference in panicle length’s MPH was significant at the heading stage.

### 2.2. Mapping Short Reads to the Rice Genome

To perform the transcriptomes of the panicle tissue in hybrid ZZY10 and the parents at the heading date, cDNA libraries were constructed, and RNA-Seq was performed on the tissue of rice panicles using the Illumina Nova-Seq platform. We obtained more than 581 million short reads for rice varieties with the three biological replicates, which aligned with gene expression levels at 0.81 < *R*^2^ < 0.98 (Figure S1). The raw data have been uploaded into the NCBI database SRA (PRJNA934656). We aligned the short reads with the Nipponbare reference genome (NCBI build 4.0), and our results showed that exonic regions covered 89.54–91.76% of reads, intronic regions covered 2.87–3.34%, and intergenic regions covered 5.30–7.17% (Table S1). In our RNA-Seq analysis, 49,044 annotated transcripts were identified in the Nipponbare reference genome (Table S2). From these annotated transcripts, we finally confirmed 9000 differentially expressed genes from the heading stage panicles (Table S3). We identified a total of 18,503 novel transcript isoforms in ZZB, Z7, and ZZY10 (Table S4).

GOSlim term analysis of the novel transcripts revealed that novel genes involved in biological processes (≥6%), followed by metabolic processes (≥4%), organic substance metabolic processes (≥3%), cellular processes (≥3%), primary metabolic processes (≥3%), cellular metabolic processes (≥3%), macromolecule metabolic processes (≥2%), cellular macromolecule metabolic processes (≥2%), macromolecule metabolic processes (≥2%), and single-organism processes (≥2%) represented approximately 30% of the novel transcripts (Figure S2). In the molecular function category, more than 40% of the novel transcripts were classified as molecular function (≥11%), binding (≥7%), catalytic activity (≥6%), heterocyclic compound binding (≥4%), and organic cyclic compound binding (≥4%) (Figure S2). A total of 40% of novel transcripts belonged to cellular components (≥12%), membrane (≥7%), cell (≥6%), cell parts (≥6%), intracellular (≥5%), and intracellular parts (≥5%) of the cellular component GOSlim categories (Figure S2). The number of novel transcripts that were categorized in biological processes was the highest compared to the others.

### 2.3. Differentially Expressed Genes (DEGs) Identified by RNA-Seq

**Figure 1:**
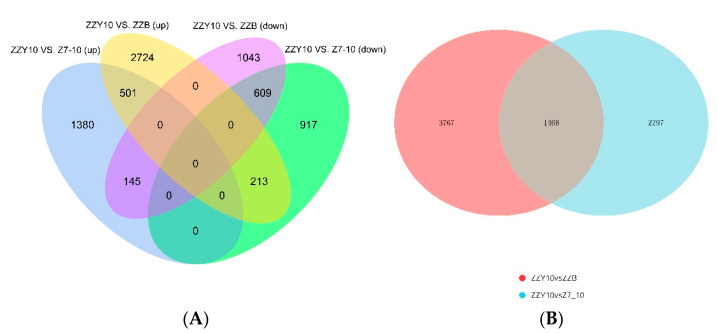
Venn diagram of DG_HP_. (**A**) Number of up-regulated and down-regulated genes expressed in two cross combinations; (**B**) Number of genes expressed in two cross combinations.

FPKM (Fragments Per Kilo bases per Millionreads) involves the influence of sequencing depth and gene length on read count. Since three biological replicates of the experiments were performed, we used DESeq2 software to analyze the differentially expressed genes and used a *p* value less than or equal to 0.05 as the standard of a significant difference. Based on these criteria, 9000 reliable DEGs were identified among the three genotypes. DEGs between the hybrid ZZY10 and its two parents, ZZB and Z7-10, were designated as DG_HP_. Considering that different expression between the hybrid and its parents is the underlying basis of their different phenotypes, DG_HP_ could be relevant to the phenotypes of heterosis [23]. Comparative transcriptome analysis revealed that a total of 5235 transcripts, including 3438 up-regulated and 1797 down-regulated transcripts, were between ZZY10 and ZZB (Figure 1A). A total of 3765 transcripts (2026 and 1739 transcripts commonly up-regulated and down-regulated, respectively) were between ZZY10 and Z7-10 and were differentially expressed (Figure 1A). In addition, 1468 transcripts between ZZY10 vs. Z7-10 and ZZY10 vs. ZZB were commonly regulated (Figure 1B). More transcripts showed differences between ZZY10 and ZZB. These results showed that the gene expression profile of ZZY10 is more similar to that of Z7-10 than that of ZZB. Principal component analysis demonstrated that ZZY10 is much closer to Z7-10 (Figure S3). Moreover, the transcriptome profile of ZZY10 was similar to Z7-10 from the hierarchical cluster analysis (Figure S4). These results consistently demonstrated that ZZY10 is closer to Z7-10 than ZZB.

**Figure 2:**
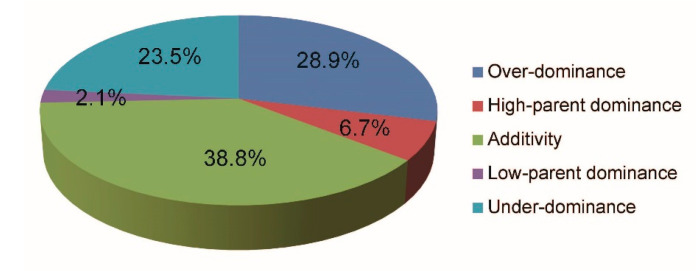
Breakdown of the DG_HP_ according to the expression pattern.

When comparing the expression levels of all the DEGs in ZZB, Z7-10, and ZZY10, we found a composite expression pattern, including five sub-patterns: over-dominance, additivity, low-parent dominance, high-parent dominance, and under-dominance, which suggested multiple modes of action (Table 3). The percentages of up-regulated, additivity, and down-regulated genes in DG_HP_ are 35.6%, 38.8%, and 25.6%, respectively. As shown in Figure 2 and Table 3, the distributions of DG_HP_ are over-dominance (28.9%), high-parent dominance (6.7%), additivity (38.8%), low-parent dominance (2.1%), and under-dominance (23.5%). First, the majority of DG_HP_ exhibits additivity. Second, the percentages of over-dominant DG_HP_ and under-dominant DG_HP_ are 28.9% and 23.5%, respectively (Figure 2 and Table 3). Finally, the percentages of additivity, over-dominance, and under-dominance account for 91.2% of all DG_HP_ (Figure 2 and Table 3). 

### 2.4. Gene Ontology (GO) Analysis

**Figure 3:**
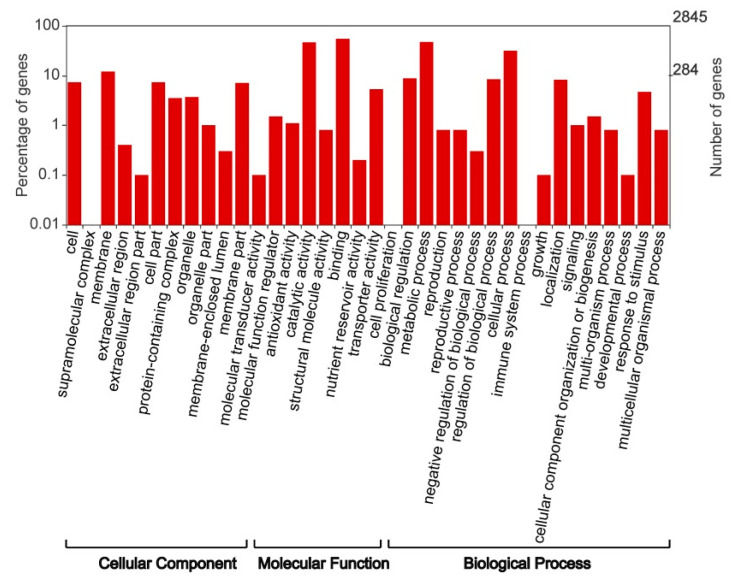
Comparison of GO term classifications of DG_HP_ at the heading stage.

GO enrichment analysis was performed for the functional classification of DG_HP_ to better understand their roles in different biological activities. In total, 2845 of the 9000 DG_HP_ between the hybrid and its parents were assigned to at least one term among the categories of cellular component (CC), biological process (BP), and molecular function (MP). Transcripts were further divided into 36 subcategories to provide an overview of ontology content (Figure 3). In the MP category, the most represented groups were binding and catalytic activity. In the CC category, the membrane and cell were prominently represented, while metabolic processes and cellular processes dominated the BP category.

We further identified that DG_HP_ were significantly enriched in GO terms (FDR-corrected *p*-values < 0.05) (Table 4). GO terms such as photosynthesis, DNA integration, cell wall modification, thylakoid, photosystem, and photosynthetic membrane were significant, suggesting that these significant pathways may be related to panicle length. 

### 2.5. Kyoto Encyclopedia of Genes and Genomes (KEGG) Enrichment Analysis

**Figure 4:**
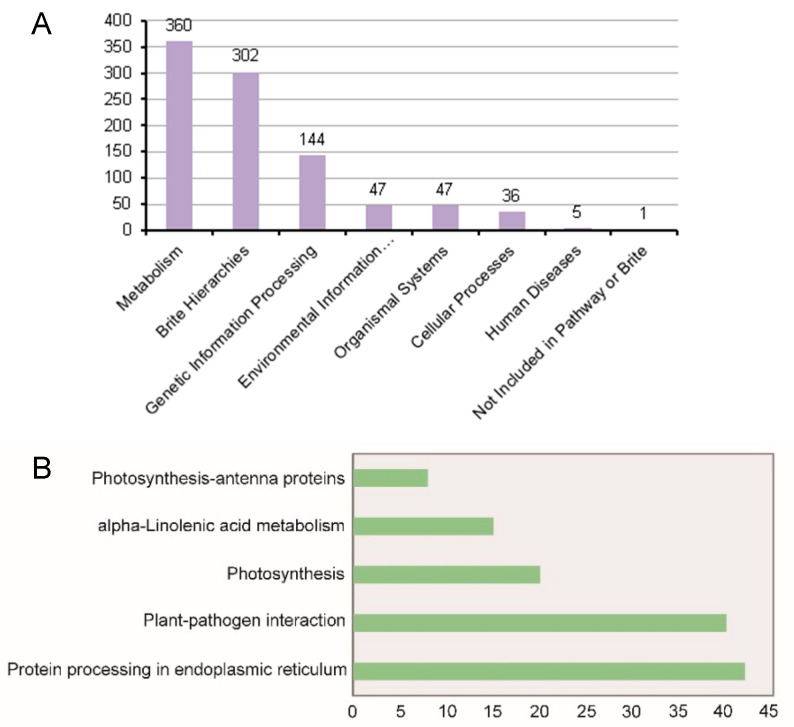
(**A**) KEGG pathway assignments into eight functional categories. (**B**) The significant enrichment list of KEGG pathways in subcategories. The significantly enriched pathway (*p* < 0.05) and the number of enriched transcripts in each pathway are shown. The x-axis in Figure B indicates the number of differentially expressed genes of these terms that are significantly enriched.

To identify metabolic pathways in which DEGs between the hybrid and its parents were enriched, we performed a KEGG analysis. Our results demonstrated that 584 of the 9000 DG_HP_ were classified into eight functional categories and were mainly involved in the pathways of brite hierarchy, metabolism, genetic information processing, cellular processes, environmental information processing, and organismal systems (Figure 4). These eight categories were further categorized into 97 subcategories corresponding to their functions, and the significant DG_HP_-involved KEGG Orthology (KO) terms were identified, including photosynthesis, plant-pathogen interaction, alpha-linolenic acid metabolism, photosynthesis-antenna proteins, and protein processing in the endoplasmic reticulum. These annotations may supply a useful resource for investigating specific processes, pathways, and functions related to heterosis of panicle length.

### 2.6. Quantitative Real-Time PCR (qRT-PCR)

**Figure 5:**
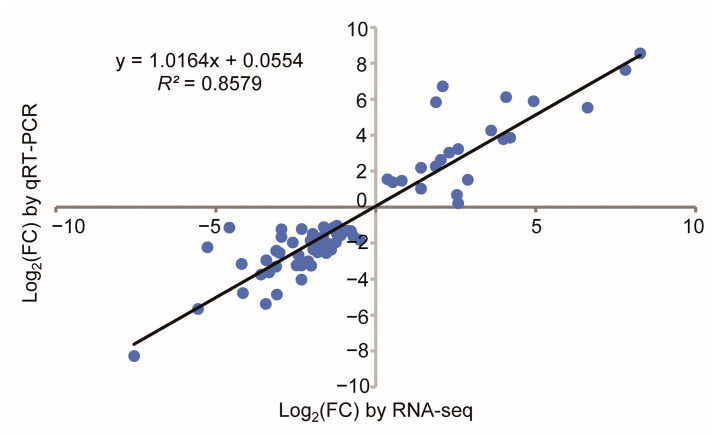
Association analysis on 38 selected transcripts between RNA-Seq and qRT-PCR.

To prove the reliability of the transcriptome results, we selected a subset of 38 DG_HP_ that was used for qRT-PCR validation. We randomly selected 17 DG_HP_, and the other DG_HP_ were pointed at photosynthesis. Primer sequences are displayed in Table S5. The results of the transcriptome and quantitative PCR were analyzed. Our comparison results showed that the expression of transcripts obtained from qRT-PCR and RNA-Seq had consistent trends (*R*^2^ = 0.8579) (Figure 5).

## 3. Discussion

Heterosis, which has been widely applied in crop breeding, plays an important role in crop production, though its functional mechanisms must still be clarified. DG_HP_ that causes different phenotypes could be related to heterosis [23,28,29,30,31]. In this study, our RNA-Seq analysis demonstrated that 581 million high-quality short reads were generated from panicle tissues of ZZY10 and its parents at the heading stage, and 49,044 annotated transcripts were aligned in the Nipponbare reference genome (Tables S1 and S2). Transcriptional profiling of F_1_ hybrid rice ZZY10 and its parents by serial analysis of gene expression revealed 9000 DG_HP_, among which DG_HP_ were found significantly enriched in pathways such as photosynthesis, DNA integration, cell wall modification, thylakoid, and photosystem. Two of the key genes involved in the photosynthesis pathway exhibited up-regulated expression in F_1_ hybrid rice. Additionally, 38 DG_HP_ of RNA-Seq were verified by qRT-PCR.

### 3.1. Comparative Analysis of the Annotated DG_HP_

A subset of DG_HP_ was observed in our comparative transcriptome analysis at the heading stage. Among the significant DG_HP_-involved GO terms, the top five significant GO terms were photosynthesis, DNA integration, cell wall modification, thylakoid, and photosystem, respectively. In the two significant pathways, 21 DG_HP_ were detected in the photosynthesis pathway and 16 DG_HP_ in the photosystem pathway. 2 DG_HP_ both in the photosynthesis pathway and in the photosystem pathway were up-regulated in the F_1_ hybrid. In this study, the panicle length of ZZY10 showed mid-parent heterosis at the heading stage. Interestingly, transcriptional expression levels of PsbP (Os12g0564400) in ZZY10 are higher than Z7-10 and lower than ZZB, which is consistent with the phenotype of panicle length in ZZY10. The PsbP proteins are a key component of photosystem II in eukaryotic photosynthetic organisms [32]. The functional characterization of PsbP homologs revealed that *Arabidopsis* mutants play important roles in maintaining photosynthetic electron transfer [33]. It can be assumed that DG_HP_ involved in the photosynthesis pathway may regulate the heterosis of panicle length.

### 3.2. Photosynthesis Regulation in Heterosis

**Figure 6:**
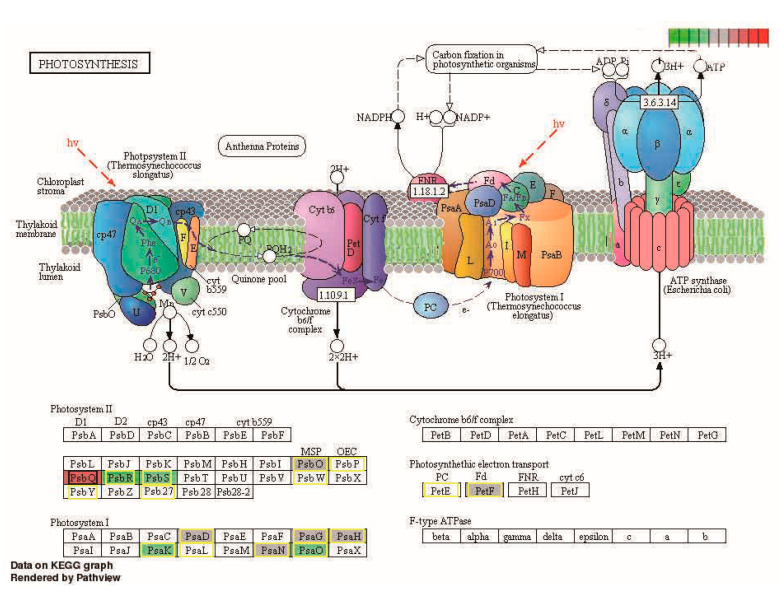
KEGG enrichment pathway of differential expression genes in the photosynthetic system. Black and yellow boxes indicate up-regulated and down-regulated genes, respectively.

Photosynthetic electron transport complex contains two light-energy-driven photosystems (PSI and PSII), the ATP synthase, and the cytochrome (Cyt) b6f [32]. By comparative analysis of transcriptomes, significantly different photosynthesis was observed in the panicle tissues of the hybrid and the parents (Figure 6). Many DG_HP_-encoded proteins involved in photosynthesis have complex organizations (Figure 6). Oxygenic photosynthesis generates molecular oxygen via the water-oxidizing reaction under the catalysis of PSII, which consists of intrinsic and extrinsic subunits of membrane proteins [34,35]. In this regard, transcripts encoding PsbO, PsbP, PsbR, PsbS, PsbW, PsbY, and Psb27, which are subunits of PSII (Os01g0501800, PsbO; Os07g0141400, PsbP; Os07g0147500 and Os08g0200300, PsbR; Os01g0869800 and Os04g0690800, PsbS; Os01g0773700, PsbW; Os08g0119800, PsbY and Os03g0333400, Psb27), were down-regulated in ZZY10 compared with the parental lines. However, Os02g0578400 and Os07g0105600, which encode PsbQ, were more up-regulated in ZZY10 than the parental lines. The high level of PsbQ expression in ZZY10 may play a role in photosynthesis and promote panicle length.

In the linear electron transport (LET), the water-extracted electrons in PSII are delivered to PSI via Cyt b6f and eventually generate NADPH. Transcriptional expression levels of PsaD (Os08g0560900), PsaG (Os09g0481200), PsaH (Os05g0560000), PsaK (Os07g0148900), PsaL (Os12g0420400), PsaN (Os12g0189400), and PsaO (Os04g0414700), which are subunits involved in photosystem I (PSI), were down-regulated in ZZY10. Two DG_HP_ (Os06g0101600 and Os03g0835900) that encode PetE and PetF in the photosynthetic electron transport showed down-regulated expression in ZZY10. The up-regulated PsbQ, down-regulated subunits, and photosynthetic electron transport in the photosynthesis pathway observed in our study may co-regulate the panicle length of ZZY10. It can be assumed that photosynthesis is involved in the heterosis of panicle length, but a more accurate characterization will be done in the future to prove it.

### 3.3. Implications for the Mechanism of Heterosis

The detection of multiple expression patterns of DEGs, such as under-dominance, additivity, and over-dominance (Figure 2), suggested that heterosis is a complex issue. It is difficult for a single hypothesis to verify its molecular basis since additivity hypotheses, dominance, or over-dominance could all contribute to rice heterosis [36,37]. Among the 9000 DG_HP_, 3536 are down-regulated and 5464 are up-regulated. In total, 3492 (38.8%), 2601 (28.9%), and 2115 (23.5%) DG_HP_ showed additivity, over-dominance, and under-dominance, respectively. In this study, we found that most of the DG_HP_ displayed additivity. The number of over-dominant DG_HP_ was almost equal to the number of under-dominant DG_HP_. Since ZZB and Z7-10 have some excellent minor and dominant genes, respectively, ZZY10 is a large panicle hybrid rice variety that has excellent minor and dominant genes. More than half of the DEGs differ between ZZY10 and ZZB (58.2%), while less than half show differences between ZZY10 and Z7-10 (41.8%). This result could explain why the panicle phenotype of ZZY10 resembles Z7-10 more than ZZB. Therefore, the hybrid vigor of ZZY10 could be produced by beneficial allele combinations of its parents, which could complement the relatively weaker alleles, or it could also come from the accumulation of multiple minor genes between two parents.

## 4. Materials and Methods

### 4.1. Plants and Growing Conditions

ZZY10, ZZB, and Z7-10, which are hybrid rice, a maintainer line, and a restorer line, respectively, obtained in our breeding used in this study. Before sowing, rice seeds of ZZY10, ZZB, and Z7-10 were placed in the electrothermal dryer at 35 °C for 5 days and soaked in distilled water (37 °C, 2 d) to germinate before being finally sown in the fields of Fuyang, Hangzhou, and Zhejiang on May 20, 2022 (30°08 N, 119°92 E, and an altitude of 10 m). Thirty days later, 40 day seedlings of ZZY10, ZZB, and Z7-10 were transplanted into the paddy field. Panicles were collected with nine replicates to evaluate heterosis. Finally, three panicles of each genotype at the heading date were stored in −80 °C refrigerators until RNA-Seq analysis.

### 4.2. Agronomic Trait Evaluation

In this study, several agronomic traits, including panicle length, primary branch number, plant height, secondary branch number, 1000-grain weight, tiller number, grain length, and grain width, were measured according to the protocols previously described [38]. HPH and MPH were calculated as follows:

HPH = (F_1_ − HP)/HP × 100% (1)

and

MPH = (F_1_ − MP)/MP × 100% (2)

F_1_, HP, and MP stand for the performance of the first hybrid generation, the best parent’s performance, and the average performance of the parents, respectively.

### 4.3. Transcriptome Analysis

Transcriptome data was analyzed through reference genome mapping of clean reads by Bowtie2 and Tophat2 tools [39]. DESeq2 software was used to analyze DEGs. DEGs were identified using the difference significance criteria of FDR ≤ 0.05 and an estimated absolute log2 (FC) ≥ 1. GO function enrichment analysis and KEGG pathway enrichment analysis for DEGs were performed using the clusterProfiler tool. Cluster analysis was performed by Cluster 3.0 software. Annotations for differentially expressed genes were obtained from the Rice Genome Annotation Project on 8 October 2022 (http://rice.plantbiology.msu.edu/ accessed on 1 January 2020).

### 4.4. qRT-PCR

Total RNA was extracted from the panicle tissues of ZZY10, ZZB, and Z7-10. RNA of the three samples was reverse transcribed into double-stranded cDNA with a PrimeScript™ RT reagent kit with a gDNA Eraser kit (TaKaRa Bio, Ohtsu, Japan). SYBR-based qRT-PCR (Takara, Japan) was carried out with the SYBR^®^ Premix Ex Taq™ II kit (Takara Bio, Ohtsu, Japan) and a LightCycler 480 system. The reaction procedure was as follows: 95 °C for 30 s, 95 °C for 30 s, and 60 °C for 30 s (40 cycles). The results of the relative quantification experiments were analyzed by the ∆Ct method. The rice gene *ACTIN1* was used as an internal control.

## 5. Conclusions

We systematically investigated the global transcriptomes of the heading-stage panicles from the hybrid rice ZZY10 and its parents using RNA-seq. The results of GO and KEGG analysis and the heterosis functional mechanism exploration create a valuable resource for rice breeding and reveal putative transcripts that could contribute to panicle length. Expression changes of candidate genes could provide helpful information for future studies assessing possible mechanisms of panicle heterosis.

## Figures and Tables

**Table 1:** MPH and HPH of panicle traits at the heading date.

	ZZB	Z7-10	ZZY10	HPH (%)	MPH (%)
PL (cm)	26.95 ± 0.82	29.83 ± 0.94	31.45 ± 1.61	−0.77	10.79 **
1000-GW (g)	1.54 ± 0.03	1.69 ± 0.05	1.94 ± 0.04	15.03 **	20.34 **
PBN	14.89 ± 0.93	13.87 ± 0.78	14.36 ± 1.01	−2.24	1.16
SBN	53.22 ± 5.78	66.56 ± 5.55	59.22 ± 8.53	−11.02 *	−1.11
TN	25 ± 3.16	11 ± 2.83	13 ± 1.79	−47.20 **	−26.67 **
PH (cm)	123.36 ± 1.55	123.02 ± 3.43	146.07 ± 1.15	16.81 **	18.66 **
GL (cm)	9.62 ± 0.27	9.24 ± 0.20	9.55 ± 0.09	−0.80	0.80
GW (cm)	2.32 ± 0.06	2.62 ± 0.09	2.42 ± 0.07	−7.49 **	−1.88

** Significant difference with *p* < 0.01; * Significant difference with *p* < 0.05. MPH and HPH stand for mid-parent heterosis and high-parent heterosis, respectively. PL, 1000-GW, PBN, SBN, TN, PH, GL, and GW refer to panicle length, 1000-grain weight, primary branch number, number of secondary branches, tiller number, plant height, grain length, and grain width, respectively. ± means standard deviation (SD).

**Table 2:** MPH and HPH heterosis of panicle length during male gametogenesis.

Stage	ZZB (cm)	Z7-10 (cm)	ZZY10 (cm)	HPH (%)	MPH (%)
meiosis stage	11.05 ± 0.46	12.03 ± 0.41	13.84 ± 0.58	15.07	19.98
uninucleate stage	13.65 ± 0.61	14.94 ± 0.38	15.70 ± 0.48	5.11	9.85
bicellular stage	20.29 ± 0.86	21.08 ± 0.51	24.03 ± 0.69	14.03	16.19
tricellular stage	23.94 ± 0.67	26.58 ± 0.50	28.33 ± 0.72	6.56	12.13
Heading stage	26.95 ± 0.82	29.83 ± 0.94	31.45 ± 1.61	−0.77	10.79 **
Ripening stage	29.84 ± 1.50	29.76 ± 0.97	30.23 ± 1.14	−3.05 *	1.44

** Significant difference with *p* < 0.01, * Significant difference with *p* < 0.05, MPH and HPH stand for mid-parent heterosis and high-parent heterosis, respectively. ± means standard deviation (SD).

**Table 3:** DEGs between the hybrid ZZY10 and its parents.

Genetic Class	Subclass	Expression Pattern	*p*-Value (Total Number of DEGs)	
			0.05 (9000)	
			Number	%
Up-regulated	Over-dominance	ZZY10 > Z7 > ZZB	1261	14.1
		ZZY10 > ZZB > Z7	1329	14.8
		ZZY10 > ZZB = Z7	0	0
	High-parent dominance	ZZY10 = ZZB > Z7	365	4.1
		ZZY10 = Z7 > ZZB	236	2.6
Additivity	Additivity	ZZB < ZZY10 < Z7	2587	28.7
		Z7 < ZZY10 < ZZB	910	10.1
Down-regulated	Low-parent dominance	ZZY10 = ZZB < Z7	58	0.6
		ZZY10 = Z7 < ZZB	139	1.5
	Under-dominance	ZZY10 < ZZB < Z7	731	8.1
		ZZY10 < Z7 < ZZB	1384	15.4
		ZZY10 < ZZB = Z7	0	0

**Table 4:** Top ten significant GO terms of DG_HP_.

Category	GO_Term	GO_Term_Annotation	*p* Value
BP	GO:0015979	photosynthesis	2.98 × 10^−6^
BP	GO:0015074	DNA integration	5.93 × 10^−5^
BP	GO:0042545	cell wall modification	0.000219
CC	GO:0009579	thylakoid	6.08 × 10^−6^
CC	GO:0044436	thylakoid part	6.08 × 10^−6^
CC	GO:0009521	photosystem	1.31 × 10^−5^
CC	GO:0034357	photosynthetic membrane	2.04 × 10^−5^
CC	GO:0009522	photosystem I	0.000582
CC	GO:0009523	photosystem II	0.000645
CC	GO:0005618	cell wall	0.001305

BP and CC stand for biological process and cellular component, respectively.

## Data Availability

Not applicable.

## Supplementary Materials

The following supporting information can be downloaded at: https://www.mdpi.com/article/10.3390/plants12061309/s1, Figure S1: Pearson correlation of gene expression from biological replicates in ZZB, Z7-10, and ZZY10; Figure S2: GOSlim term analysis in BP, MF, and CC; Figure S3: PCA of the hybrid ZZY10 and its parents; Figure S4: The cluster analysis of all differentially expressed transcripts in ZZB, Z7-10, and ZZY10; Table S1: Summary of filtered, mapped, and reference-based assembled read data; Table S2: RPKM of annotated transcripts; Table S3: List of differentially expressed genes; Table s4: List of novel transcripts; Table S5: Primers for qPCR.
