# Supplementary material for: Transcriptomic Analysis Reveals Panicle Heterosis in an Elite Hybrid Rice ZZY10 and Its Parental Lines
Source: Plants (Basel). 2023 Mar 14;12(6):1309. doi: 10.3390/plants12061309 (PMC10059593; doi:10.3390/plants12061309)
Supplement: Supplementary file 1 [file plants-12-01309-s001.zip › Supplementary Material-plants.docx]

**Table S1**. Summary of filtered, mapped, and reference-based assembled read data.

|  | Total filtered reads | Mapped reads  (%) | Exon | % | Intron | % | Intergenenic | % |
| --- | --- | --- | --- | --- | --- | --- | --- | --- |
| ZZB | 67359206 | 88.49 | 8151902168 | 91.76 | 261851964 | 2.95 | 470525009 | 5.30 |
|  | 70334464 | 86.56 | 8218794044 | 90.63 | 303073499 | 3.34 | 547041665 | 6.03 |
|  | 73875122 | 88.66 | 8914118929 | 91.29 | 298338497 | 3.06 | 551679979 | 5.65 |
| Z7-10 | 70604774 | 87.24 | 8236784358 | 89.75 | 288865946 | 3.15 | 651782484 | 7.10 |
|  | 60853428 | 88.48 | 7274102849 | 90.66 | 230305508 | 2.87 | 518737680 | 6.47 |
|  | 61966318 | 88.39 | 7371723733 | 90.31 | 260175566 | 3.19 | 530353944 | 6.50 |
| ZZY10 | 59795444 | 85.21 | 6824740158 | 89.95 | 244689157 | 3.23 | 517595306 | 6.82 |
|  | 60678742 | 85.54 | 6977490437 | 90.25 | 227971669 | 2.95 | 525807908 | 6.80 |
|  | 55817568 | 86.13 | 6412118264 | 89.54 | 234998549 | 3.28 | 513671524 | 7.17 |

**Table S5**. Primers for qPCR.

| RAP Locus | Forward primer | | Reverse primer | | Description | | Log2(FC) ZZY10 versus Z7-10 | | | | Log2(FC) ZZY10 versus ZZB | |
| --- | --- | --- | --- | --- | --- | --- | --- | --- | --- | --- | --- | --- |
|  |  | |  | |  | RNA-Seq | | | qRT-PCR | RNA-Seq | | qRT-PCR |
| Os01g0254000 | AAGATCCTCTTCCTCGGCCT | CGACCTTGGCGTAGTAGTCC | | GTP-binding protein SAR1A | | | | -7.56 | -8.29 | -3.59 | | -3.76 |
| Os01g0757500 | TTCAGCTGGGATCAGGATGC | TGTGCAGCTTAGGTATGGCA | | calcyclin-binding protein | | | | -3.44 | -5.38 | 2.87 | | 1.51 |
| Os01g0850900 | CGCTGAGATCGAATCCGACCTT | TACTTGCGGACCTCGTAGCC | | heme-binding-like protein At3g10130, chloroplastic | | | | -4.16 | -4.78 | -2.60 | | -1.98 |
| Os02g0731600 | AACAAGTCCACCGTCCTCAC | ACGAGCTCCATGAACTTGGG | | protein CURVATURE THYLAKOID 1A, chloroplastic | | | | 7.80 | 7.62 | 3.60 | | 4.26 |
| Os02g0764100 | GTTACGTGCTGACCAAGGGA | CCGCCGTGTTGTTCTTCTTG | | B3 domain-containing protein | | | | 2.03 | 2.62 | 0.81 | | 1.46 |
| Os02g0782500 | GTGGACATCATGGAGACCCC | CGCCATTGCTGCTCTTCATC | | 18.6 kDa class III heat shock protein | | | | 4.93 | 5.89 | 3.99 | | 3.77 |
| Os03g0100200 | AGCTGTTCCTCATCGCCAT | CCACCACGCTCTTTTGCTTC | | probable pterin-4-alpha-carbinolamine dehydratase, chloroplastic | | | | 4.07 | 6.11 | 4.20 | | 3.86 |
| Os03g0266900 | ACGCAAAGCAAACCAAGCAA | GAGAAGGGGTCGAACACGTT | | 17.4 kDa class I heat shock protein | | | | -5.27 | -2.24 | -2.95 | | -1.24 |
| Os03g0626700 | TCATCCTTGTCGGCCTCCTA | CATTGAAGCCCAGTGCAACC | | protein DETOXIFICATION 29 | | | | 1.88 | 5.83 | -1.88 | | -2.40 |
| Os03g0822200 | CGAGTGCTGTGCCAAAGATG | TTGCCTAGGCTGTTCAGTGG | | uncharacterized protein At5g02240 | | | | 2.09 | 6.71 | -1.84 | | -1.91 |
| Os04g0538000 | GGAGCAATGCCTGAAGGTCT | GCAATTCCTCCTGGCTCAGT | | hsp70-Hsp90 organizing protein | | | | -2.95 | -1.67 | -2.33 | | -4.03 |
| Os05g0161200 | CTTGCATGTGGCCATCGATG | GCTGCAGCCAACATGATAGC | | GTP diphosphokinase CRSH1, chloroplastic (precursor) | | | | -2.32 | -1.23 | -3.12 | | -3.31 |
| Os05g0460000 | GCCATGAACCCAACCAACAC | ACCACCTTGAACGGCCATAG | | probable mediator of RNA polymerase II transcription subunit 37c | | | | 8.26 | 8.55 | -5.56 | | -5.67 |
| Os06g0228200 | TTACGTCGATCTTCGCAGGG | AAGTAGATCCAGAGGCCGGT | | Low silicon protein 6, NOD26-like intrinsic protein 2-2 | | | | 2.53 | 0.67 | 1.87 | | 2.26 |
| Os07g0412100 | GAGGACTTGTCGACACGGTT | CTTGGAATGCCGGTGTGTTG | | granule-bound starch synthase 1b, chloroplastic/amyloplastic | | | | 6.62 | 5.53 | -4.58 | | -1.14 |
| Os08g0440100 | AGGTCAAGTTCTACGTGCCC | CGTACACCTCCTCGTCCATG | | temperature-induced lipocalin-1 | | | | -1.56 | -1.25 | -1.65 | | -1.66 |
| Os07g0105600 | TTTGCCGGTGCCAAAGATTG | CTTGAGGCGGAGGTACTTGG | | photosynthetic NDH subunit of lumenal location 3, chloroplastic | | | | 2.57 | 0.21 | 1.41 | | 1.02 |
| Os12g0564400 | AACAACGAGCTGGCGGTGAT | GGAGCTCGTACAGCCGCTTG | | Similar to Thylakoid lumenal 21.5 kDa protein, chloroplast precursor | | | | 0.36 | 1.54 | -0.85 | | -1.32 |
| Os01g0501800 | GAGACCGGCGAGGTGATTGG | ACATCCTTGGGCACCTTGGC | | oxygen-evolving enhancer protein 1, chloroplast precursor, putative, expressed | | | | -1.22 | -1.04 | -2.12 | | -3.02 |
| Os07g0141400 | AGCGTAACGGTGCTGACGAG | GCGCCCTTGAACCACCTCTT | | Similar to 23 kDa polypeptide of photosystem II | | | | -0.94 | -1.33 | -1.69 | | -2.24 |
| Os07g0147500 | GGCTGCCTCTGTCATGGCTT | ACTCCTCCAGCAGGCCCATA | | photosystem II 10 kDa polypeptide, chloroplast precursor, putative, expressed | | | | -3.12 | -2.45 | -3.43 | | -2.96 |
| Os08g0200300 | GTCTGCAGCGGTGGGAAGAA | TGTCACCGGTGGGAGACCAT | | photosystem II 10 kDa polypeptide, chloroplast precursor, putative, expressed | | | | -1.49 | -2.05 | -1.96 | | -1.67 |
| Os01g0869800 | GCTGTTCGGCAGGTCCAAGA | TGGCTTCGGCTCAGCCTTTC | | 22-kDa Photosystem II protein | | | | -1.10 | -1.57 | -1.96 | | -2.36 |
| Os04g0690800 | GCATGGCGATGCCGATGATG | CCGAAGATGCCGTCCTCCAC | | 22-kDa Photosystem II protein | | | | -3.11 | -2.43 | -1.40 | | -2.37 |
| Os01g0773700 | GGGTGTTCGGCCTCATCTGG | CCGGACTCCTCGTCCTCCTC | | photosystem II reaction center W protein, chloroplast precursor, putative, expressed | | | | -1.25 | -1.96 | -2.03 | | -3.26 |
| Os08g0119800 | GGCCATGCTTAAGCCGTCCA | AGCGAGATGCTAGGCACCCT | | photosystem II core complex proteins psbY, chloroplast precursor, putative, expressed | | | | -0.78 | -1.33 | -1.63 | | -1.12 |
| Os03g0333400 | GCCATCGCCGTAAAGCCAAC | CGTCCTTCGTCTCGCTCACG | | photosystem II 11 kD protein, putative, expressed | | | | -4.20 | -3.16 | -1.54 | | -2.32 |
| Os02g0578400 | ACCTCGACCTCGACCTCACC | GTCCGCACGAACCGATCGAA | | oxygen evolving enhancer protein 3, identical, putative, expressed | | | | 2.29 | 3.02 | 0.52 | | 1.38 |
| Os07g0105600 | CCGACAGGGCCAACTACGTC | GAGCCGATCTGGGTGTCGTC | | oxygen evolving enhancer protein 3 domain containing protein, expressed | | | | 2.57 | 3.22 | 1.41 | | 2.18 |
| Os08g0560900 | ACCAGGCTCCGCTCCAAGTA | GGGCTGACGTTCTTGCCGAT | | photosystem I reaction center subunit II, chloroplast precursor, putative, expressed | | | | -1.32 | -1.13 | -1.84 | | -2.05 |
| Os09g0481200 | GAGAACGTGGCGAAGCAGGT | ACGTCGACGAGGTTGAAGCC | | photosystem I reaction center subunit, chloroplast precursor, putative, expressed | | | | -1.61 | -2.43 | -2.33 | | -3.24 |
| Os05g0560000 | CGTCGCCGTACAACCCTCTC | CTGCGGGCCCTTCTTGATCG | | expressed protein | | | | -1.23 | -1.45 | -1.97 | | -1.49 |
| Os07g0148900 | TCCATGGTGACGCTGCCATC | CATCAGCGTCGTCGTCGTCA | | photosystem I reaction center subunit, chloroplast precursor, putative, expressed | | | | -2.42 | -2.68 | -3.34 | | -3.64 |
| Os12g0420400 | CCTTCATCGGCAGCCTGGAG | GCCGGGAGGTTGGAGAGGTA | | photosystem I reaction center subunit XI, chloroplast precursor, putative, expressed | | | | -1.16 | -1.42 | -1.82 | | -2.53 |
| Os12g0189400 | CAAGAAGAGGCTGGCGACGA | CTGGCAGCCGGTGAAGTTGT | | photosystem I reaction center subunit N, chloroplast precursor, putative, expressed | | | | -2.04 | -1.87 | -3.01 | | -2.55 |
| Os04g0414700 | GATCGGGTTCGGGCTGATCG | CCTGGCCGATGCTGGAGAAG | | membrane protein, putative, expressed | | | | -2.48 | -3.25 | -3.10 | | -4.86 |
| Os06g0101600 | CAAGAACAACGCCGGCTTCC | GCGTGACGGAGAAGGTCTCG | | Plastocyanin, chloroplast precursor | | | | -0.70 | -1.64 | -1.55 | | -2.57 |
| Os03g0835900 | AGCCTGAAGGCCTCCTCCAA | CCAGCACGGCATGAGTAGGG | | Similar to Ferredoxin III, chloroplast precursor (Fd III) | | | | -1.07 | -1.43 | -0.51 | | -1.83 |

RAP Locus is the gene IDs starting with Os. FC is fold change which represents the ratio of expression quantity between two samples. The absolute value of log2(FC) greater than 1 was the screening criterion for differential genes.


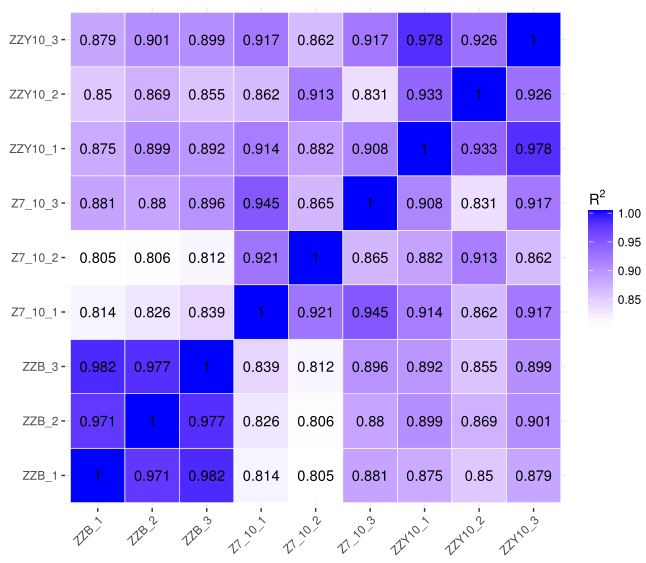


**Figure S1**. Pearson correlation of gene expression from biological replicates in ZZB, Z7-10, and ZZY10.

**Figure S2**. GOSlim term analysis in BP, MF, and CC.

BP, MF, and CC stand for biological process, molecular function and cellular component, respectively.


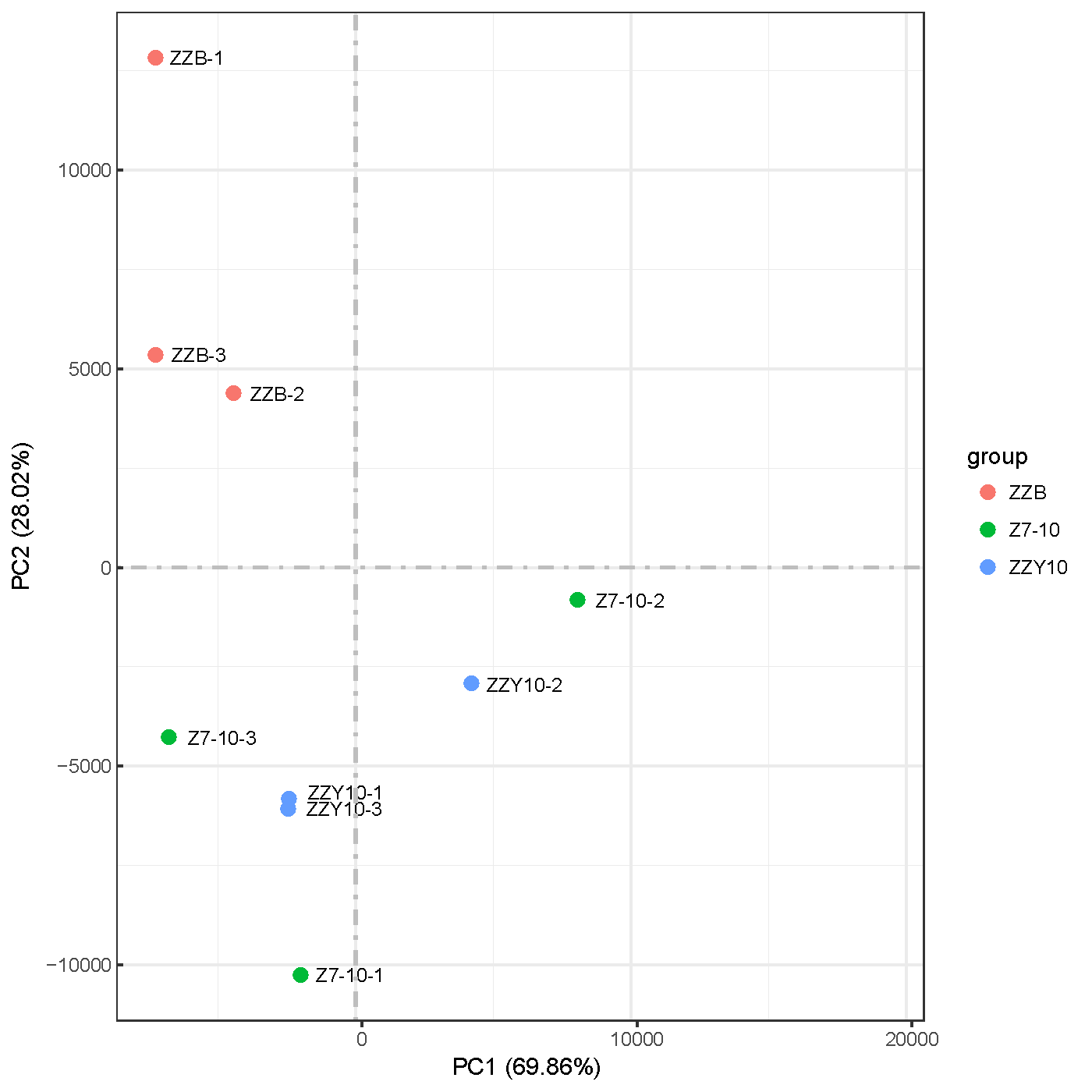


**Figure S3**. PCA of the hybrid ZZY10 and its parents.


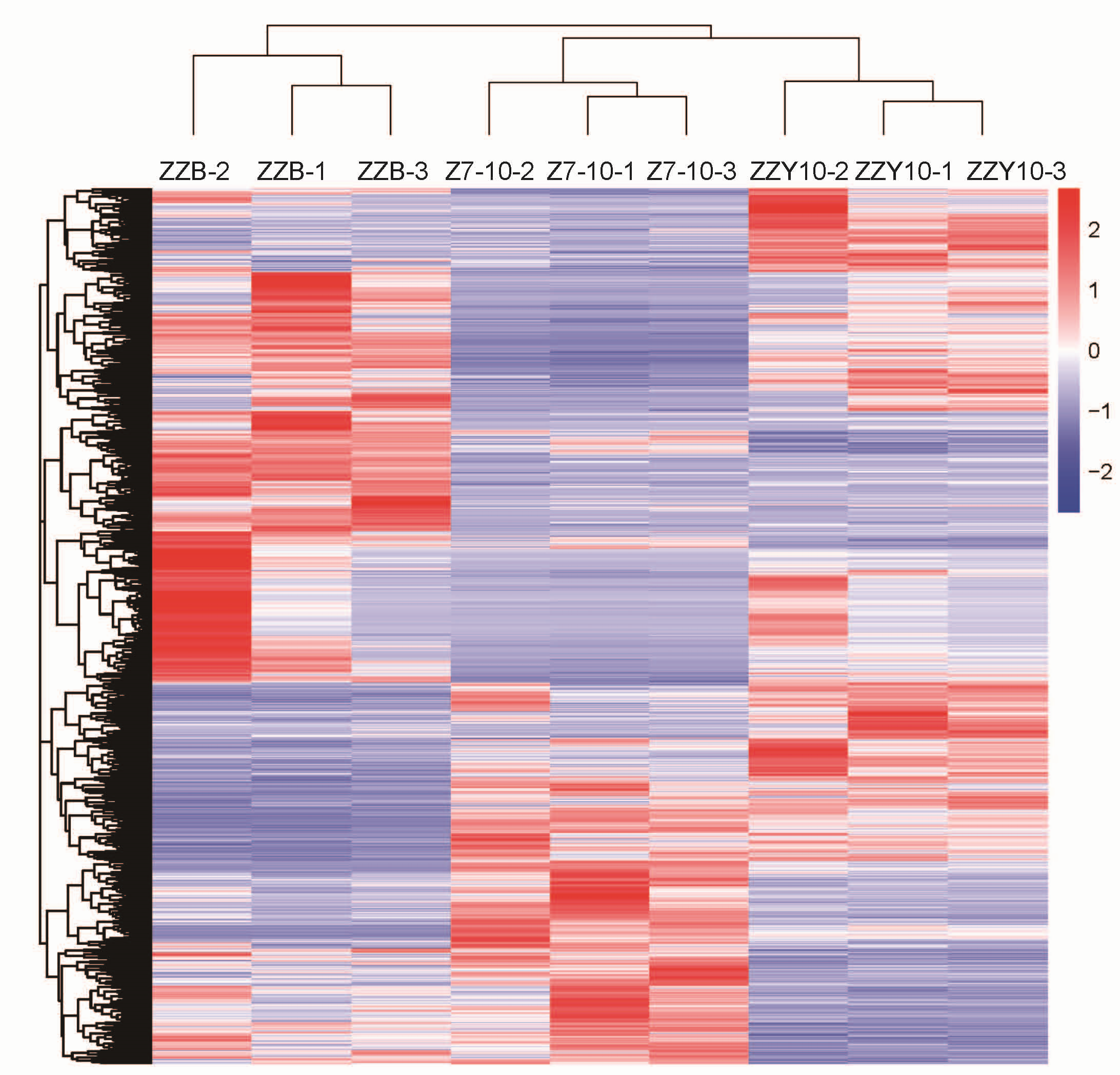


**Figure S4**. The cluster analysis of all differentially expressed transcripts in ZZB, Z7-10, and ZZY10. Red and blue represents high and low expression, respectively.
